# A novel platform simulating irregular motion to enhance assessment of respiration‐correlated radiation therapy procedures

**DOI:** 10.1120/jacmp.v6i1.2058

**Published:** 2005-03-17

**Authors:** Mathew J. Fitzpatrick, George Starkschall, Peter Balter, John A. Antolak, Thomas Guerrero, Christopher Nelson, Paul Keall, Radhe Mohan

**Affiliations:** ^1^ Department of Radiation Physics The University of Texas M. D. Anderson Cancer Center Houston Texas 77030; ^2^ Department of Radiation Oncology The University of Texas M. D. Anderson Cancer Center Houston Texas 77030; ^3^ Department of Radiation Oncology Virginia Commonwealth University Richmond Virginia 23298 U.S.A.; ^4^ Present address: Radiation Oncology Mayo Clinic Rochester MN 55905 U.S.A.

**Keywords:** 4D imaging, computed tomography, respiratory motion, respiration‐correlated radiation therapy

## Abstract

Respiratory motion continues to present challenges in the delivery of radiation therapy to tumors in the thorax and abdomen by causing movement of structures within those areas. Several approaches to account for this movement in the planning and delivery of treatment have been developed over the past several years. To assist in the development and assessment of various techniques for respiration‐correlated radiation therapy, a platform capable of programmable irregular longitudinal motion has been designed and fabricated to simulate intrafractional respiratory motion. A sliding platform and the base on which it was mounted were constructed from polycarbonate plastic, and a stepper motor provided platform motion. Respiratory motion data, either artificially generated on a spreadsheet or extracted from respiratory monitoring files, were converted to a format appropriate for driving the stepper motor. Various phantoms were placed on top of the platform and used in studies related to respiration‐correlated radiation therapy. Several applications of the platform were demonstrated, such as improving the quality of acquisition of time‐dependent computed tomography image datasets, comparing various methods of acquiring such datasets, and implementing feedback‐guided breath hold treatment delivery procedures. This study showed that a platform capable of programmable irregular motion is a useful tool for the development and assessment of procedures related to the effects of respiratory motion in radiation therapy.

PACS number: 87.66.Xa

## I. INTRODUCTION

Respiratory motion continues to present challenges in the delivery of radiation therapy to tumors in the thorax and abdomen by causing movement of structures within those areas. Several approaches to account for this movement in the planning and delivery of treatment have been developed over the past several years. Some of these methods developed by our group and others include gating the delivery of radiation during specified phases in the respiratory cycle^(1)^; delivering radiation during patient breath hold, either voluntary^(2)^ or assisted^(3)^; and allowing the patient to breathe freely while explicitly determining the extent of respiratory motion from a series of computed tomography (CT) image datasets acquired at various phases in the respiratory cycle.^(4)^ Moreover, new techniques for implementing respiration‐correlated radiation therapy, such as our recently reported feedback‐guided breath hold,^(5)^ are continually being developed. While these techniques are under development, it is necessary to simulate various aspects of respiration to demonstrate the clinical practicality of such techniques.

Concurrent with the development of respiration‐correlated planning and delivery techniques is the development of techniques for acquiring CT image datasets that accurately depict respiratory motion.^(^ ^6^ ^–^ ^10^ ^)^ These techniques, known as four‐dimensional (4D) CT, involve monitoring periodic respiratory motion, acquiring image information at corresponding phases in the respiratory cycle using a multislice helical CT scanner, and reconstructing and collating the image information into image datasets, with each set representing a single phase in the respiratory cycle. Commercial manufacturers of CT scanners use various strategies for acquisition of this phase‐dependent image information. As these systems are being developed, it is necessary to have appropriate tools to assess the new technologies for acquisition of 4D CT image datasets and to determine the optimal acquisition parameters for these techniques.

In addition to respiration‐correlated radiation therapy and 4D CT image acquisition, approaches are being developed to account for respiratory motion in radiation dose calculations.^(11)^ ^,^ ^(12)^ To validate the accuracy of a 4D dose calculation algorithm by comparison with measurements, it is necessary to simulate respiratory motion so that radiation doses can be measured on a moving phantom.

To achieve all of these goals, it is necessary to develop a means of simulating respiratory motion, enabling an appropriately designed phantom to move with a variety of periodic, quasi‐periodic, and irregular motions. Although most of the previous development of respiration‐correlated imaging and treatment techniques has been done with phantoms capable of sinusoidal motion, real patients do not breathe sinusoidally.^(13)^ Their respiratory cycles are somewhat irregular, even with visual biofeedback. Moreover, patients whose respiration has been compromised may have very irregular respiratory cycles. Robust technologies for implementing various aspects of respiration‐correlated radiation therapy must be able to account for such respiratory motions. Consequently, it would be highly desirable to test respiratory correlation techniques using a phantom capable of being programmed with various kinds of motions that simulate the variety of patient breathing patterns encountered in the clinical use of respiration‐correlated radiation therapy. To this end, we have developed a platform capable of programmable irregular motion. Here, we describe the design and fabrication of this platform and illustrate some applications of its use in respiration‐correlated radiation therapy.

## I. MATERIALS AND METHODS

### A. Movable platform

The irregular breathing (IrrB) platform was designed to be able to simulate the gross anatomical superior‐inferior motion attributable to respiration‐induced motion of the thoracic region. Depending on the particular application, an appropriate phantom would be placed on the platform. Examples of application‐specific phantoms might include a CT imaging phantom to assess image quality of 4D imaging techniques and an anthropomorphic phantom to evaluate the dosimetric impact of respiratory motion. The construction of the platform was inspired by the work of Vedam and Keall^(14)^; however, we have improved on their design by adding the capability to program irregular motion free of sinusoidal regularities.

**Figure 1:**
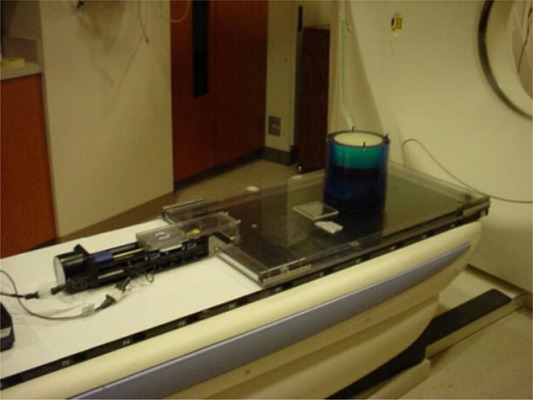
The movable IrrB platform

The essential component of the device is a movable platform mounted to a base using precision linear bearings (Fig. 1). The base and platform are made of clear, 12.7‐mm thick polycarbonate plastic, and the linear ball bearings are oriented to restrict the platform to a displacement of approximately 5 cm along a straight line with very little friction. The platform is driven along a linear trajectory using a motor controller that drives a stepper motor attached to the BiSlide assembly (Velmex, Inc., Bloomfield, NY). The motor controller can be programmed to simulate most types of respiratory motion, including actual patient respiration; the use of a BiSlide with a lead screw pitch of 2 mm per revolution reduces the amount of inertial torque that a heavy load can impress directly upon the motor. Programs are stored as text files on a personal computer and can be downloaded to the motor controller through a serial RS‐232 interface.

Stepper motors are capable of highly accurate positioning and are generally adequate for systems that operate at low accelerations with static loads. A motor positioning system with no displacement feedback and a simple open‐loop control system must be validated to ensure that the motor system is not subject to excess torque. Because a stepper motor is electromagnetically driven and has no gears, subjecting the system to excess torque without feedback can cause a loss of rotor positioning control (i.e., slippage) and therefore a loss of platform positioning control. This is more of a problem when the motion system requires sudden acceleration or variable loads, whether real or effective, that might occur in 2D or 3D systems. The linear bearings alleviate this problem to some extent.

A BiSlide with a smaller pitch also helps to reduce the torque that a stepper motor must apply to move a load; however, the stepper motor must then turn at a higher number of revolutions per second to compensate for the resulting decrease in displacement per revolution. Also, as the rotational speed of the stepper motor increases, the torque that the stepper motor can apply without slippage decreases, so a balance must be struck for the positioning needs.

### B. Programming the stepper motor

The initial file used to simulate respiratory motion was generated in a spreadsheet (Excel, Microsoft Corp., Redmond, WA), either by use of a mathematical generating function or extracted from a patient's respiratory trace monitored by an external fiducial tracking system (RPM, Varian Medical Systems, Palo Alto, CA). The fiducial tracking system outputs data in time‐displacement pairs. The displacement is reported as the distance from the top of the screen by the fiducial tracking system. Thus the displacement data is multiplied by ‐1 and then, for convenience, shifted to center the entire dataset about the *x*‐axis. We then assumed that the set of time‐displacement pairs correlated with superior‐inferior diaphragm motion.^(13)^ The motor controller simulates a 1D trajectory by running in continuous mode.

**Figure 2:**
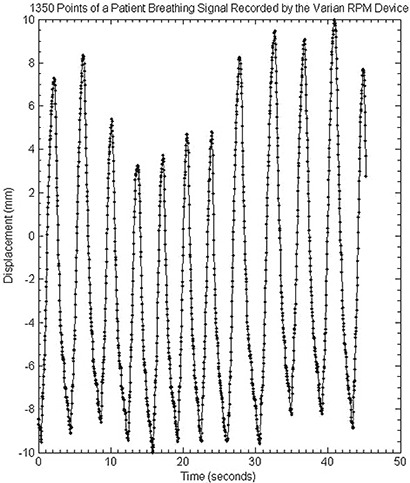
Example of a patient's respiratory trace extracted from the RPM respiratory‐monitoring device

The present commercial release of the stepper motor controller was designed to be driven by repetitive or short motion profiles, not the entire respiratory trace typically encountered in a 4D CT imaging session. Figure 2 is an example of a typical respiratory trace. In normal use, a driving program is loaded into the controller's limited memory, and once execution begins, no new trajectory information can be downloaded. Trajectory information is communicated to the stepper motor system by passing a set of velocity‐displacement data pairs,

$$\left(v_{i},\Delta y_{i}\right)=\left(\frac{\Delta y_{i}}{\Delta t},\Delta y_{i}\right),$$

in which the displacement, Δ*y*, is expressed as a number of steps (1 step=0.0050 mm for our motion system), and the velocity, *v,* is given in steps per second.

To compensate for the limited memory of the controller, we reduced the data consisting of approximately 1350 data pairs $\left(t_{i},\;y_{i}\right)$ produced by the respiratory monitoring program by dividing the dataset into a smaller number of intervals. Within each interval, we approximated the *n* data pairs with a second‐order polynomial,

$$\begin{array}{ccc} y(t)=at^{2}+vt+y_{0}, & & t_{i}<t<t_{i+n}, \end{array}$$

using a nonweighted linear least‐squares approximation. The velocity‐distance data pair was found by taking the average velocity over the interval along with the change in the interval. Thus, the interval $\Delta y_{i}$. is expressed by

$$\Delta y_{i}=y(t_{i+n})-y(t_{i})=[a_{i}(t_{i+n}+t_{i})+v_{i}](t_{i+n}-t_{i})$$

Then

(1) $$(v_{i},\Delta y_{i})=\left(\frac{y(t_{i+n})-y(t_{i})}{t_{i+n}-t_{i}},y(t_{i+n})-y(t_{i})\right)$$

**Figure 3:**
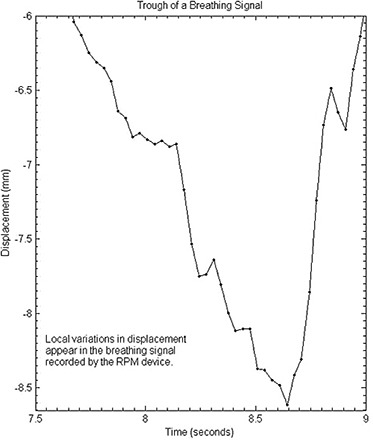
Detailed view of a respiratory trace illustrating small irregularities in the trace

Because the respiratory‐monitoring program produces sampled data pairs approximately every 30 ms, local variations in amplitude, $Y_{i}$ appeared as seen in Fig. 3, perhaps due to noise. These local variations need to be eliminated because they can be too impulsive to be modeled by the motor and could cause slippage. The least‐squares fitting smoothed these local noise variations.

**Figure 4:**
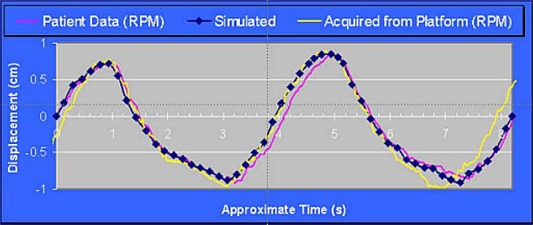
Comparison of the motion of the IrrB platform predicted on the basis of a file extracted from the RPM device and the actual motion of the IrrB platform

The use of a second‐order polynomial simplified algorithm prototyping and development. In addition, straightforward linear least‐squares fitting seemed to de‐emphasize some of the local curvature seen in respiratory signals over large intervals. The second‐order polynomial appeared to be sufficiently robust to catch the velocities associated with the curvature seen in respiratory signals, for example, at the minimum and maximum amplitudes, while still successfully smoothing out the signal over local data pairs. For the profile used in Fig. 4, the speed varied between 0.4mm/s and 20 mm/s, with an average speed of 8 mm/s. Although it was not pursued in this research, further efficiency in data reduction should be possible if the intervals are segmented into positive slope, negative slope, and extremum regions.

The positioning system is not an idealized mechanical system, so some latency occurs in any trajectory program. To force the period of a respiratory trajectory to comply with our needs, we scaled the velocity, the only parameter with time in it, by a constant factor, α, so that the previous equation could be better written as

(2) $$\left(\alpha v_{i},\Delta y_{i}\right)=\left(\alpha \left(\frac{y(t_{i+n})-y(t_{i})}{t_{i+n}-t_{i}}\right),y(t_{i+n})-y(t_{i})\right)$$

Finally, despite great care in smoothing the data, impulsive changes in velocity over small displacements may occur. For example, for our design a change in speed above 500–700 steps/s within an interval of less than 50 steps caused slippage. Inspection of the trajectory is then necessary, and manual intervention is required to reduce the impact of impulsive values that may cause slippage.

## C. Applications

One potential application of the IrrB platform is to improve the quality of 4D CT image acquisition. The image acquisition approach used by one commercial vendor (Philips Medical Systems, Cleveland, OH) involves the phase binning of projections in the sinogram file before reconstruction.^(7)^ ^,^ ^(15)^ Initial studies of this approach identified specific circumstances in which the quality of the 4D CT image dataset was not up to clinically acceptable standards, particularly for slow breathing and certain types of irregular breathing cycles. Using a phantom placed on the IrrB platform programmed to move with appropriate breathing patterns enabled us to provide feedback to the vendor to help improve their technique for 4D CT image acquisition.

A second commercial vendor (General Electric Medical Systems, Waukesha, WI) has developed an alternative approach to 4D CT image acquisition in which CT images are acquired in axial mode and retrospectively binned on the basis of a record of the patient's respiratory cycle.^(6)^ With the aid of the IrrB platform, we have imaged a CT quality assurance phantom (Catphan 500, The Phantom Laboratory, Salem, NY) under a variety of respiratory configurations to compare the two approaches to 4D CT image acquisition.^(16)^

In a third application of the IrrB platform, we have developed a technique for delivering radiation while the patient is executing a feedback‐guided breath hold maneuver.^(5)^ In developing this procedure, it was necessary to simulate regular respiration, breath hold, and deviations from breath hold, such as coughing, demonstrating that the delivery of radiation would occur only while the patient is appropriately executing the breath hold and that radiation would not be delivered during any breath‐hold deviations. Such respiratory patterns were relatively simple to program into the IrrB platform, which provided a useful tool to assist in the development of this method.

## III. RESULTS

Although the respiration record is readily produced in the form of a spreadsheet indicating the position of the platform as a function of time, the files input to the stepper motor have to be converted into pairs specifying the velocity and duration of velocity. Consequently, slight deviations exist between the initial respiratory motion and the motion of the IrrB platform. An example of a comparison of the actual motion of the IrrB platform to the predicted motion is illustrated in Fig. 4. As shown by the figure, deviations were negligible and did not interfere with the performance of the IrrB platform.

**Figure 5:**
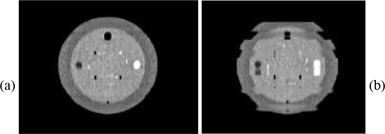
CT images of the test phantom extracted from a 4D CT image dataset acquired at (a) 20 breaths/min and (b) 10 breaths/min

Because respiratory rates could be programmed into the IrrB platform, we were able to investigate the role of the respiratory rate in the quality of 4D CT images acquired using the approach of phase‐binning projections acquired in helical mode. Figure 5 compares CT images of the phantom acquired at respiratory rates of (a) 20 breaths/min and (b) 10 breaths/min. Both image datasets were acquired on a 16‐slice helical CT scanner (MX8000‐IDT, Philips Medical Systems, Cleveland, OH) using a 0.5‐s gantry rotation time and a pitch of 0.125. Severe image artifacts were observed at 10 breaths/min. These artifacts were caused by the motion of the CT table being too rapid for the respiratory cycle, resulting in acquisition of an inadequate number of projections for reconstruction. Because of these findings, a recommendation was made to the vendor to provide slower table translation, an idea that has since been implemented.

**Figure 6:**
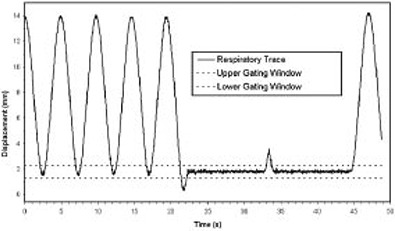
Respiratory trace generated by the IrrB platform used to validate feedback‐guided breath hold radiation delivery

Finally, Fig. 6 shows the respiratory trace generated by the IrrB platform programmed to simulate regular breathing for several cycles, followed by a breath hold, followed by a cough, followed by another breath hold. This profile was used to demonstrate that radiation was delivered only during the breath hold and not during normal respiration or during the cough. Using the IrrB platform, we were able to develop and test the ability of the LINAC to deliver radiation only during a breath hold and not during the normal respiratory cycle or during interruptions of a breath hold.

## IV. DISCUSSION

Before the development of the IrrB platform, demonstrations of respiration‐correlated imaging and treatment delivery techniques were limited to what could be developed using regular sinusoidal motion to simulate respiration. Some aspects of respiration‐correlated imaging and treatment delivery were expressed only when respiratory motion was irregular and consequently could not be accurately reproduced in phantom studies. As demonstrated by several examples, we have found that the IrrB platform is a useful tool for assessing and developing techniques to account for respiratory motion. The IrrB platform enables the simulation of various forms of respiratory motion, including motion extracted from a commercial respiratory monitoring device, thus simulating the respiratory motion of actual patients. Several potential applications of the IrrB platform were reported to illustrate use of the platform. Full details of the applications will be addressed in other reports.

Several limitations presently exist with the IrrB platform. It currently simulates only 1D motion. The design of the platform to simulate 1D motion was predicated on the need to simulate irregular motion in as simple a framework as possible. Most of the issues that are needed to be addressed in 4D imaging can be expressed adequately by simulating only 1D motion. Moreover, most of the present systems used to monitor respiratory motion (e.g., external fiducials, spirometers, strain gauges) are 1D. Phantoms are placed on the movable platform; consequently, motion exhibited by the phantoms in this study are rigid and nondeforming. Nevertheless, the IrrB platform has a relatively simple design, is easy to construct and use, and has the potential for many applications in the development and clinical implementation of respiration‐correlated radiation therapy.

## ACKNOWLEDGMENTS

The authors wish to acknowledge the assistance of Keith Daniel and Pat Burnett in the fabrication of the platform. We also wish to thank Brian Crow of Philips Medical Systems for his expert service of the multislice helical CT scanner and educational discussions. Finally, we wish to thank Mitchel Evans, Cliff Nichols, and David W. Engdahl of Velmex, Inc., for their expert assistance in choosing and developing the stepper motor system.

This work was supported in part by a Sponsored Research Agreement with Philips Medical Systems and National Cancer Institute grant R01 93626.
